# Bridging Policy and Practice in Telemedicine Follow-Up Identification: Multicenter Mixed Methods Study in Beijing

**DOI:** 10.2196/75964

**Published:** 2025-12-19

**Authors:** Yanli Lyu, Xi Li, Huixian Li, Mingyu Gu, Xuedong Xu, Yipei Wang, Changxiao Jin

**Affiliations:** 1 Office of Internet Hospital, Peking University Third Hospital, Beijing, China; 2 Department of Medical Affairs, Peking University Third Hospital, Beijing, China; 3 Beijing Municipal Center for Healthcare Quality Management and Improvement in Internet-Based Medical Services, Beijing, China; 4 Institute of Hospital Management, Peking University Third Hospital, North Huayuan Road 49, Beijing, 100191, China, 86 010 82266191; 5 Party Committee Office and Dean’s Office, Peking University Third Hospital, Beijing, China

**Keywords:** assessment process, Beijing, China, follow-up visit, health policy, management, mixed methods, prerequisite, quality, telemedicine services

## Abstract

**Background:**

Telemedicine services have been developing rapidly worldwide. Following the 2018 policy enabling telemedicine for follow-up patients, this service model has gradually gained popularity in China. However, little has been done to understand the policy’s implementation across different types of medical institutions or to evaluate its effectiveness.

**Objective:**

This study aims to (1) investigate the patient eligibility assessment process in various types of institutions for telemedicine services in Beijing, (2) elucidate institutions’ rationale for adopting such approaches, (3) analyze discrepancies between policy and practice, and (4) provide references for the development of telemedicine services.

**Methods:**

This mixed methods study involved 36 medical institutions in Beijing, determined based on placing in the top 20% for both service volume and service quality in 2023. The study was conducted in 2 phases. First, quantitative analyses were conducted based on the questionnaires collected from each institution’s contact to gather information about the assessment process and patient prerequisites. Subsequently, qualitative analyses were conducted through thematic analysis of 36 semistructured interviews with each institution’s contact to acquire their considerations of this practice.

**Results:**

These 36 institutions contributed 968,786 telemedicine visits, representing 89.5% of the total service volume in Beijing. In practice, each of the visits underwent a 2-stage eligibility assessment before the physician officially accepted the patient’s request. In the first stage, for assessment approaches, 86.2% (25/29) of the nonprofit, tertiary hospitals and 14.3% (1/7) of the private, for-profit institutions automatically assessed patient eligibility, while others did it manually. The assessment was based on the scope of previous visit location, diagnosis of previous visits, and visit interval. For the scope of visit location, 22 hospitals required prior visits to the same institution. For diagnosis, 7 hospitals required patients to have an identical diagnosis. For visit intervals, 11 hospitals required it to be within 6 months. The second stage assessment was conducted by physicians. Compared with policy requirements, nonprofit hospitals had stricter requirements for the scope of visit locations. The main reasons for these discrepancies included distrust in the medical outcomes from other institutions (19/26, 73.1%) and difficulties in handling interinstitutional medical disputes (18/26, 69.2%). In addition, 61.1% (22/36) of the institutions indicated that terminations of telemedicine services were primarily due to the patient’s conditions.

**Conclusions:**

This pioneering multicenter, mixed methods study delineated the patient eligibility assessment process for telemedicine services in Beijing. Discrepancies were identified between real-world practice and regulatory prerequisites. The key factors contributing to these variations included the ambiguity of policies and different priorities across institution types. Our findings suggest greater policy clarity, relaxation of regulations for new patients, and strengthened oversight of telemedicine services to improve the quality and accessibility of telemedicine.

## Introduction

### Background

Telemedicine effectively connects individuals and their health care providers when in-person care is not necessary or not possible. Catalyzed by the pandemic, this novel service model has rapidly expanded and gained broad acceptance [1-3]. Currently, telemedicine is widely implemented in countries such as the United States, Canada, and Australia, with coverage rates reaching 35%‐60% [4-6]. In March 2020, the Centers for Medicare and Medicaid Services in the United States further facilitated adoption by removing the originating site requirement, allowing patients to receive telemedicine services from home [7]. Supported by such policy changes, telemedicine has been particularly useful in stroke treatment, psychiatry, radiology, and other chronic diseases, especially for patients in rural areas [8-11].

Similarly, in China, telemedicine services have been developing at a rapid pace [12]. In 2018, patients with “certain” chronic or common diseases were allowed to use telemedicine for follow-up visits [13], according to the Telemedicine Diagnosis and Treatment Management Measures (trial) formulated by the National Health Commission [14]. However, online initial services, including diagnoses, prescriptions, and referrals for new patients, are still prohibited [15]. By the end of 2022, the policy allowed medical institutions to prescribe medications online via telemedicine for patients presenting COVID-related symptoms. From a safety perspective, determining patient eligibility for telemedicine has remained an important consideration in policymaking. As patient demand for telemedicine continues to evolve, policies should also be updated accordingly to address these changing demands effectively.

In China, medical institutions are primarily categorized into hospitals and other medical institutions. Hospitals are further classified by function into tertiary, secondary, and first-class levels. Tertiary hospitals serve as regional medical centers with advanced capabilities in clinical care, education, and research, offering high-level specialized care across regions. Secondary hospitals provide medical and health services to multiple communities and undertake limited education and research responsibilities. First-class hospitals focus on preventive and rehabilitative care within a single community [16]. While all types of medical institutions can provide telemedicine services, tertiary hospitals remain predominant, accounting for 61.6% of the telemedicine providers by the end of 2022 [17].

Since the implementation of the telemedicine policy for follow-up patients in 2018, domestic studies have primarily focused on the service volume, operational framework, or organizational implementation [18-20], with relatively few studies evaluating the service process from the perspective of the patient’s journey. Currently, only limited research has addressed telemedicine practice variances across different types of institutions [18,21], and extensive research is still needed on institutional implementation considerations and strategies. These research gaps hinder the timely updates and refinements of policies, hence impeding the effective delivery of telemedicine services.

To address this gap, our study investigated institutional approaches to telemedicine follow-up identification in Beijing. Although Beijing is a city with high-concentrated, high-quality health resources [22], its telemedicine services were comparable to those of other provinces and municipalities in terms of service accessibility and provision by tertiary hospitals. The adoption rate of telemedicine among hospitals in Beijing was within the range of 0.700‐0.840, placing it among the top 10 provinces in China. Similar rates were observed in Jiangsu, Shanghai, Zhejiang, Ningxia, and Xinjiang [23]. In Beijing, about 30% of the tertiary hospitals provided telemedicine services, ranking 16th among 31 provinces [17]. Therefore, Beijing is representative in terms of service volume and the dual-channel service model. Its experience in formulating telemedicine policies positions it as an emerging leader in telemedicine, providing valuable reference for other regions and countries seeking to develop and refine telemedicine policies. While regions should still tailor telemedicine strategies to their own situation and local needs, the experiences from Beijing still offer important guidance for designing region-specific policies.

### Aims and Objectives

Based on the analyses of 36 institutions, this study aimed to examine discrepancies between policy and practice in telemedicine follow-up identification. By investigating patient eligibility assessment in Beijing, elucidating institutions’ rationale for such approaches, and analyzing the deviations from the national policies, we identified the key issues, proposed strategies to improve the efficiency of telemedicine services, and highlighted potential areas for policy refinement.

## Methods

### Overview

**Figure 1.**
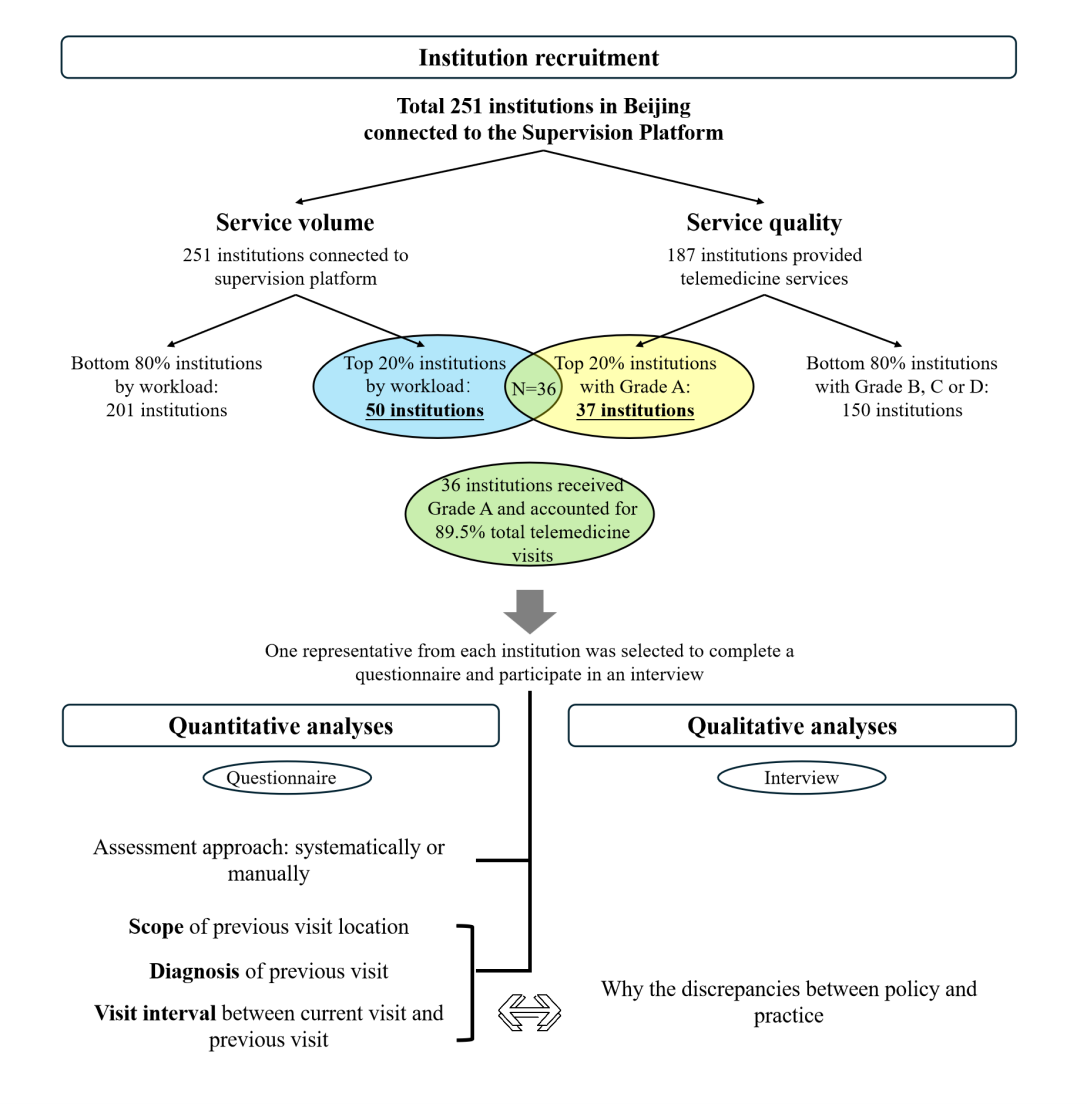
Study flow diagram. The relationship and sequence of mixed methods research components, including institution recruitment, quantitative analyses, and qualitative analyses: the light blue shade indicates the number of institutions selected due to the service volume, the light yellow shade indicates the number of institutions selected due to the service quality, and the light green shade (the intersection of the blue shade and the yellow shade) indicates the institutions recruited for this study.

The reporting of this study followed the checklist of mixed method research studies by Lee et al [24] in *Medical Care Research and Review* (Checklist 1), adopting an explanatory sequential design consisting of 3 phases: institution recruitment, quantitative analyses, and qualitative analyses (Figure 1) [25]. During the institution recruitment phase, based on the total volume and service quality of telemedicine services in 2023, 36 institutions were selected from 251 medical institutions in Beijing. Next, in the quantitative phase, we distributed survey questionnaires to these 36 institutions asking them to specify their prerequisites for providing follow-up visits across 3 dimensions, including scope of prior visit (same physician, same department, same institution, or not specified), diagnostic consistency (identical diagnosis, similar diagnosis, or not specified), and interval between visits (within 6 mo, within 1 y, within 3 y, or not specified). Then, we calculated the frequency of each option to characterize prevailing practices. Finally, in the qualitative research, semistructured interviews were conducted to explore the potential reasons for discrepancies between policy and practice in follow-up determination.

This design was grounded on 2 rationales. First, integrating quantitative and qualitative data facilitates a synergistic combination of their strengths. The quantitative phase utilized a structured questionnaire to capture the prevalence and distribution of patient eligibility assessment processes for telemedicine services. Concurrently, qualitative interviews offer insights into why the institutional prerequisites for patient assessment functioned. Second, combining quantitative surveys with qualitative interviews enabled data triangulation, thereby enhancing the credibility of our findings on patient assessment prerequisites across different institutions.

Since the study references multiple policy-related concepts, key terms are defined below to clarify the policy context and facilitate the interpretation of the assessment process:

1. Patient eligibility assessment process: This process evaluates whether patients meet the criteria for receiving telemedicine services. It comprises 2 stages: before treatment and during treatment.
2. Prerequisites for patient assessment: In the first stage of patient eligibility assessment, patient eligibility could be assessed either automatically by the system or manually by the physician. The assessment usually covers 3 dimensions, including the scope of previous visit location, diagnosis of previous visits, and visit interval.
3. Scope of previous visit location: Before addressing a patient-initiated request for a telemedicine visit, the scope of requested telemedicine care will be compared to the patient’s prior in-person visits to determine whether the encounter involves the same hospital, the same department, or the same physician.
4. Diagnosis of previous visits: Before addressing a patient-initiated request for a telemedicine visit, the chief complaint reported for the current initiated virtual care will be compared with previous in-person visits to assess whether the present diagnosis is identical to or related to the previous diagnosis.
5. Visit interval: The time interval between the current telemedicine visit and the previous in-person visit will be reviewed to determine whether it is within 6 months, within 1 year, or within 3 years.
6. Termination: If the patient’s condition does not meet the service requirements set by the proposed institution, such as violating any of the 3 dimensions described above or applying reasons, physicians could stop providing telemedicine services during either the first or second stage of the assessment process.
7. Beijing Telemedicine Service Supervision Platform (referred to as “Supervision Platform”): Developed by the Beijing Municipal Health Commission, the Supervision Platform is a centralized data aggregation platform that requires institutions to upload their telemedicine service information. The Supervision Platform can also automatically calculate the compliance monitoring scores for each institution.
8. Compliance monitoring metrics: Compliance monitoring metrics are built-in indicators within the Supervision Platform to evaluate the quality of telemedicine services. These metrics reflect situations that could lead to point deductions, including 9 qualification metrics, 17 operational metrics, and 8 supervision metrics (Multimedia Appendix 1). For all uploaded telemedicine visits, each recorded violation results in a 1-point deduction. Thus, 1 visit with several quality deficiencies can lead to multiple points deductions.
9. The Beijing Municipal Center for Healthcare Quality Management and Improvement in Internet-Based Medical Services (hereafter referred to as “the Center”): The Center is responsible for telemedicine-related oversight, such as issuing monthly statistical bulletins, conducting inspections, organizing experience-sharing sessions, and providing education and training programs. The Supervision Platform enables the Center to monitor and promptly deliver feedback to institutions.

### Institution Recruitment

Based on the total volume and quality of telemedicine services in 2023, research participants were selected from 251 medical institutions in Beijing. Regular inspections by the Center revealed that institutions with higher service volume and quality tended to maintain more consistent assessment standards. Thus, institutions with high service volume and quality were selected for the study.

The volume of telemedicine services referred to the number of telemedicine visits uploaded by medical institutions to the Supervision Platform. A total of 50 institutions ranked in the top 20% by service volume were initially considered from the perspective of service volume.

The quality of telemedicine services was determined based on compliance monitoring scores. The annual average compliance monitoring deduction per visit for each institution was calculated. Institutions were ranked in ascending order based on their scores. The top 20% (n=37) of the institutions received Grade A, indicating the presence of a standardized and efficient management system. These institutions were considered from the perspective of service quality.

We then intersected the top 20% of the institutions in service volume with those by service quality, yielding a final sample of 36 institutions (Figure 1). These 36 institutions consisted of 29 nonprofit tertiary hospitals and 7 private, for-profit institutions. Among them, the 29 nonprofit hospitals included 14 general hospitals, 11 specialized hospitals, and 4 traditional Chinese medicine (TCM) hospitals, covering all categories of tertiary public hospitals in Beijing.

These 36 institutions collectively accounted for 89.5% of the overall telemedicine service volume in Beijing, representing both nonprofit and private, for-profit institutions with high service volume and quality. Therefore, their patient eligibility assessment processes could reflect the mainstream practice of telemedicine services.

### Data Collection and Management Procedures

#### Platform-Based Data Extraction

In August 2024, the institutional information and 2023 service volume data for each of the 36 institutions were obtained from the Supervision Platform.

#### Questionnaire Survey

The approaches and prerequisites of patient eligibility assessment for telemedicine services in medical institutions were collected through questionnaire surveys conducted in September 2024. Each institution designated a contact for communication with the Center, and their demographic information was compiled in a spreadsheet. A structured questionnaire with 5 questions was designed and distributed sequentially to the contacts of all 36 institutions via Wenjuanxing (an online survey platform), with a 1-week deadline for completion. The survey questionnaire is available in Multimedia Appendix 2. Response progress and data completeness were monitored in real time through the Wenjuanxing administrator interface. Reminders were sent to nonrespondents, and incomplete submissions were flagged for follow-up to ensure data integrity.

#### Semistructured Interview

The considerations of these prerequisites for patient assessment were collected through semistructured interviews. After completing the questionnaire online, the contacts of the 36 institutions were interviewed individually via telephone calls from November 2024 to February 2025. All participants provided verbal informed consent. The semistructured interviews were conducted in Mandarin Chinese by the authors YL, XL, HL, and MG. At least 2 of them were present to conduct the interview in each session. All interviewers received professional training in qualitative interviewing and had extensive experience conducting qualitative research. The semistructured interview outline is provided in Multimedia Appendix 3. We recorded the interviews using a digital voice recorder. The interview phase concluded once interviews with all institutions were completed and frequency statistics were compiled. The interviews had an average length of 15 minutes, with an overall length of 526 minutes. Notes were taken simultaneously in Microsoft Word 2019 (Microsoft Corp.), and recordings were transcribed into textual materials within 48 hours after the interviews by the interviewers present on-site. The interviewees were allocated a randomly selected unique identification number.

### Data Analysis

Descriptive statistics were used to analyze the quantitative data using Microsoft Excel 2019 (Microsoft Corporation) and SPSS 22 (IBM Corp). The distribution of all categorical variables (eg, type of institution, gender, age, role in the department, and departmental affiliation of the interviewees) was reported as frequencies (n) with percentages (%). The service volume was reported as mean with SD and median with IQR. Frequency distribution plots were generated to visualize the distribution of prerequisites for patient assessment.

Qualitative analysis followed an inductive approach, and the data were systematized and analyzed by manual coding. All textual transcripts were imported into Excel 2019 (Microsoft Corp.). Thematic analysis was employed to generate initial codes from the qualitative data [26]. Three of the authors (YL, XL, and YW) read all of the transcripts and analyzed the data independently.

Initial manual codes were developed respectively, and data segments were assigned to these codes. The codes were clustered into broader themes, which were then merged, split, or discarded following a review of the transcripts after discussion among the above authors. The themes were renamed to accurately reflect their content, resulting in the coding framework (Multimedia Appendix 3). Two of the authors (YL and XL) conducted the coding independently. After completing the coding, both authors compared the coding selections, and when discrepancies occurred, consensus was reached through discussion with a third author (YW) until agreement was reached. The number of recurring themes was recorded and documented. A comparative analysis was performed to evaluate discrepancies between on-the-ground practices and national policy requirements, with gaps and their root causes systematically identified. Reasons for the termination of telemedicine services were further analyzed to determine contributing factors. Finally, the themes were translated into English for reporting purposes.

### Ethical Considerations

This study was reviewed and approved by the Medical Science Research Ethics Committee of Peking University Third Hospital (No. IRB00006761-M2024300). The Beijing Municipal Health Commission agreed to use the data from the Supervision Platform for secondary analysis. The interviewees provided informed consent. We filed the survey data while maintaining full confidentiality. The quantitative data were anonymized upon the conclusion of the study via a 2-step process: (1) institutions were categorized and (2) all identifying names were removed. Similarly, the qualitative data were stripped of all identifying details during the transcription process to ensure anonymity. No compensation was provided for participation.

## Results

### Characteristics of Investigated Institutions

In 2023, 187 institutions provided telemedicine services throughout the year, totaling 1,082,591 visits in Beijing. Among them, the institution with the highest service volume had 164,868 visits, while the institution with the lowest service volume had only 1 visit. The average service volume was 5789.3 visits, with an SD of 21,999.7 visits. The median service volume was 49, the 25th percentile was 13, the 75th percentile was 444, and the IQR was 431.

This research focused on analyzing 36 institutions that distributed questionnaires. The service volume of the 36 institutions can be found in Table 1. The total telemedicine service volume of the 36 institutions included in this study was 968,786, accounting for 89.5% of the total service volume in Beijing. As a result, the services provided by these 36 institutions could reflect the status of telemedicine services in Beijing. The total amount of services in 2023 of the 36 medical institutions was 968,786 (100%), with the highest annual service volume of 164,868 visits and the lowest of 220. The average service volume was 26,910.7 visits, and the SD was 42,043.8. The median of the service volume was 9709.5, the 25th percentile was 2919, the 75th percentile was 29,649.5, and the IQR was 26,730.5.

**Table 1.** Distribution of institutions and service volume, by institution type (n=36).

Type of institution	Institutions, n (%)	Number of services		
		Total, n (%)	Mean (SD)	Median (IQR)
Nonprofit	29 (80.6)	805,917 (83.2)	27,790.2 (41,031.3)	11,163 (31,740)
Tertiary general hospital	14 (38.9)	292,801 (30.2)	20,914.4 (42,644.8)	8414 (9453)
Tertiary specialized hospital	11 (30.6)	386,094 (39.9)	35,099.5 (43,317.9)	13,866 (41,308)
Tertiary TCM^a^ hospital	4 (11.1)	127,022 (13.1)	31,755.5 (33,771.8)	27,744.5 (61,829.5)
Private, for-profit	7 (19.4)	162,869 (16.8)	23,267 (49,351.7)	4125 (12,108)

a TCM: traditional Chinese medicine.

Among the 36 institutions investigated in this study, 29 were nonprofit hospitals, all of which were tertiary hospitals. They included 14 general hospitals, 11 specialized hospitals, and 4 TCM hospitals. The total telemedicine service volume of the 29 nonprofit hospitals in 2023 was 805,917 (83.2%), with an average service volume of 27,790.2 per hospital. The median service volume of 29 nonprofit hospitals was 11,163, and the IQR was 31,740.

The other 7 institutions were private, for-profit medical institutions, including 1 secondary general hospital, 2 primary TCM hospitals, 2 ungraded general hospitals, 1 first-class TCM hospital, and 1 clinic. The total telemedicine service volume of the 7 private, for-profit medical institutions in 2023 was 162,869 (16.8%), with an average service volume of 23,267 per institution. The median service volume of the 7 private, for-profit institutions was 4125, and the IQR was 12,108.

### Characteristics of Interviewees

A total of 36 contacts from 36 institutions specializing in telemedicine services were interviewed for this study (Table 2). These interviewees had experience in outpatient management, engaged in hospital administration, or were responsible for technology-related work. After the implementation of telemedicine services in these institutions, they were assigned operational roles to coordinate and maintain the services. Among the interviewees, 20 were leaders of telemedicine services, and 16 were participants familiar with the work. The average age of interviewees was 40 years.

**Table 2.** A summary of interviewees’ demographic information (n=36).

Characteristics	Interviewees, n (%)
Sex	
Male	15 (41.7)
Female	21 (58.3)
Age (y)	
30‐39	19 (52.8)
40‐49	10 (27.8)
50‐59	7 (19.4)
Role in the department	
Leader	20 (55.6)
Participant	16 (44.4)
Departmental affiliation of the interviewees	
Affiliated to the outpatient department	16 (44.4)
Independent, office of telemedicine	10 (27.8)
Independent, operation planning department	7 (19.4)
Affiliated to medical administration office	2 (5.6)
Affiliated to the information technology department	1 (2.8)

### Patient Eligibility Assessment Process for Telemedicine Services

After patients registered and updated their information on the telemedicine application or WeChat mini-program of each hospital, they could apply for telemedicine services. The institution providing telemedicine services conducted the assessment, which was divided into 2 stages. The first stage took place before treatment. In this stage, the assessment was made automatically by the system or manually by the physician. The second stage occurred during treatment. The attending physician determined whether the patient’s condition was suitable for telemedicine services. If institutions did not use the system for assessment in the first stage, the 2 stages were combined, and the assessment was left to the physicians. In each stage, specific reasons could prevent patients from receiving telemedicine services. If physicians determined that a patient was not suitable for telemedicine services, they could terminate the session and refer the patient to in-person care. The medical service fee for this session would be refunded.

The assessment approaches of the 36 investigated institutions are presented in Table 3. In the first stage, 72.2% (26/36) of the institutions made assessments automatically by the system, including 25 of the 29 tertiary nonprofit hospitals and 1 of the 7 private, for-profit medical institutions. Among the tertiary nonprofit hospitals, only 4 hospitals selected qualified patients for telemedicine services manually by the physicians, including 1 general hospital, 2 specialized hospitals, and 1 TCM hospital. These hospitals provided a total of 19,465, accounting for 2.42% of the service volume of nonprofit hospitals. Among the 7 private, for-profit medical institutions, only 1 first-class TCM hospital assessed patients automatically by the system. The other 6 private, for-profit institutions assessed patients manually by the physicians for follow-up appointments through telemedicine.

**Table 3.** Distribution of assessment approach by hospital type (n=36).

Type of hospital	Auto^a^, n (%)	Manual^b^, n (%)
Nonprofit	25 (69.4)	4 (11.1)
Tertiary general hospital	13 (36.1)	1 (2.8)
Tertiary specialized hospital	9 (25.0)	2 (5.6)
Tertiary TCM^c^ hospital	3 (8.3)	1 (2.8)
Private, for-profit	1 (2.8)	6 (16.7)

a Automatically by the system.

b Manually by the physician.

c TCM: traditional Chinese medicine.

**Figure 2.**
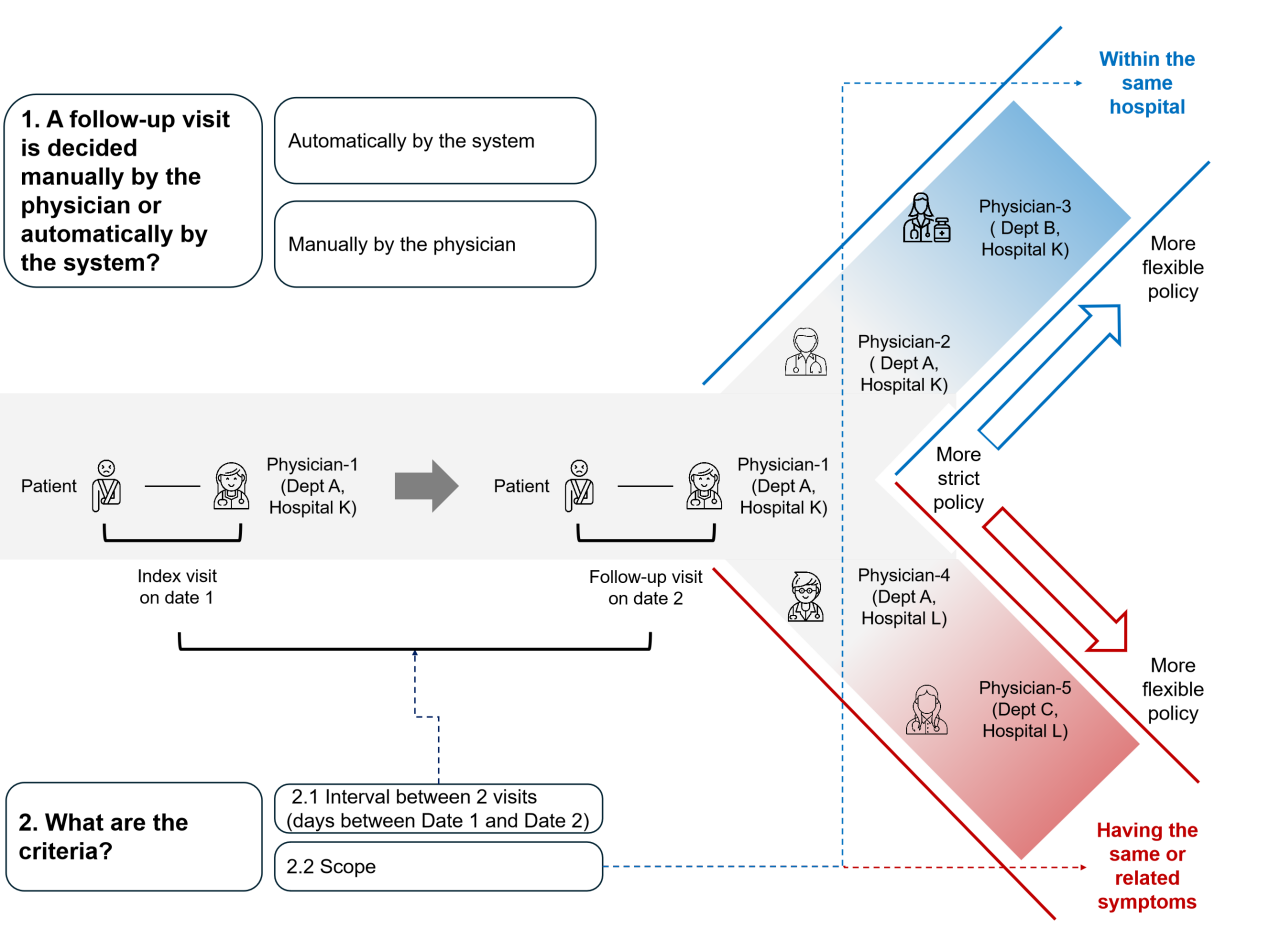
Patient eligibility assessment process for telemedicine services in the first stage.

Regardless of whether the assessment was conducted automatically by the system or manually by the physician, 36 medical institutions investigated in this study assessed patient eligibility from the following 3 dimensions in the first stage. Figure 2 illustrates the patient eligibility assessment process and the 3 dimensions: scope of previous visit location, diagnosis of previous visits, and visit interval. First, whether the previous visit location fell within the scope set by the current institution. Second, whether the previous diagnosis was within the scope defined by the current institution. Third, whether the visit interval between the patient’s previous and current visits was within the scope set by the current institution. In this study, we found that medical institutions providing telemedicine services did not necessarily set the requirements for the above 3 dimensions. Considering the ownership type of the institutions, we would discuss the 29 nonprofit tertiary hospitals and the 7 private, for-profit institutions separately.

### Prerequisites for Patient Assessment in Nonprofit Tertiary Hospitals in Beijing

#### Prerequisites for the Scope of Previous Visit Location

**Figure 3.**
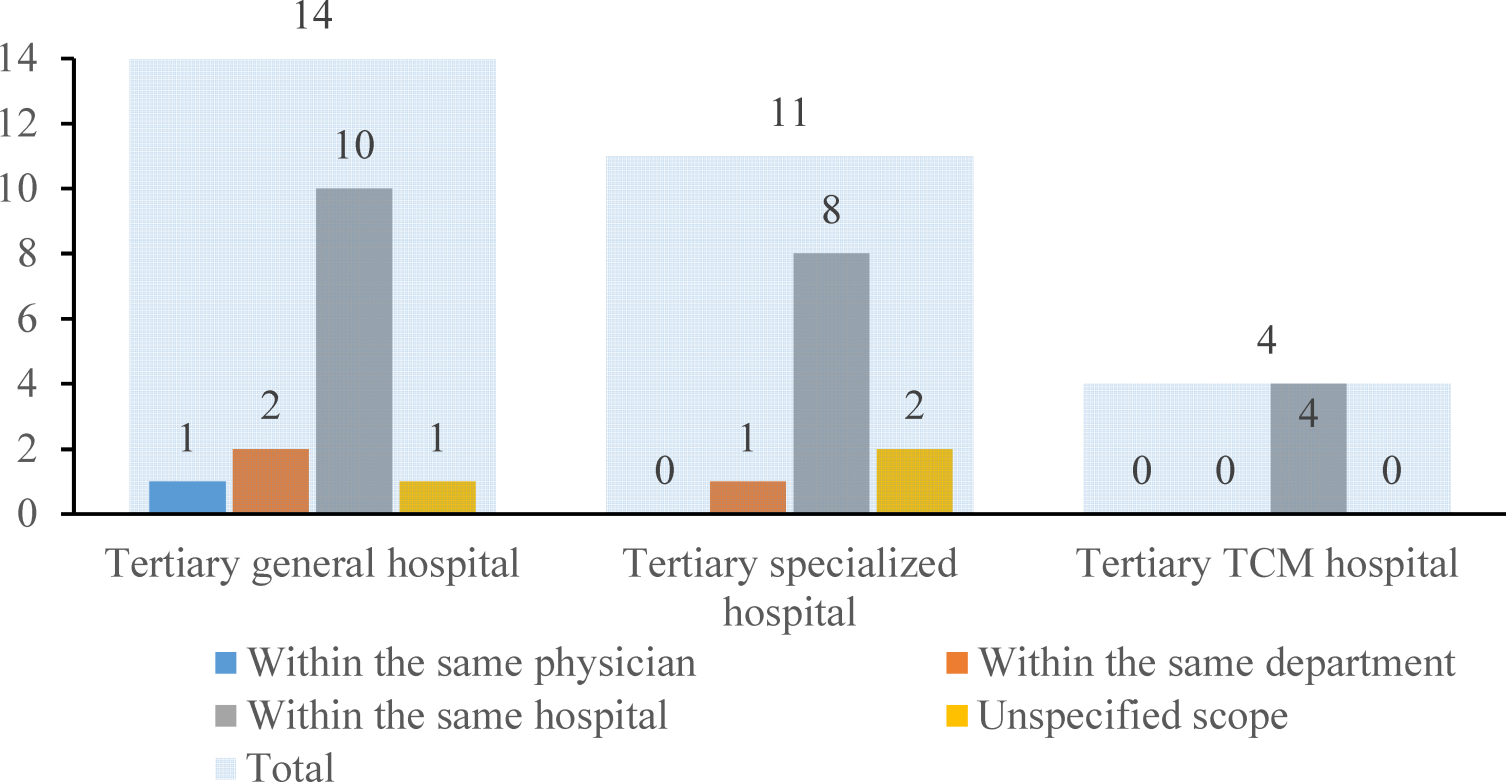
Institution distribution on prerequisites for the scope of previous visit location (n=29). The light blue shade indicates the sum in each type of hospital. The blue, orange, gray, and yellow columns indicate the number of hospitals that required the scope of the previous location to be within the same physician, the same department, the same hospital, and unspecified scope, respectively. TCM: traditional Chinese medicine.

The prerequisites for the scope of previous visit location were categorized into 4 groups: within the same physician, within the same department, within the same hospital, and unspecified scope. Figure 3 shows that 75.9% (22/29) of the nonprofit tertiary hospitals restricted prior visits to the same institution. There was 1 general hospital that required a record of previous visits to the same physician within the hospital. There were 2 general hospitals and 1 specialized hospital that required a record of previous visits to the same department within the hospital. All the TCM hospitals only limited the scope to the same hospital. Additionally, 3 hospitals did not impose any requirements regarding the previous visit location.

#### Prerequisites for Diagnosis of Previous Visits

**Figure 4.**
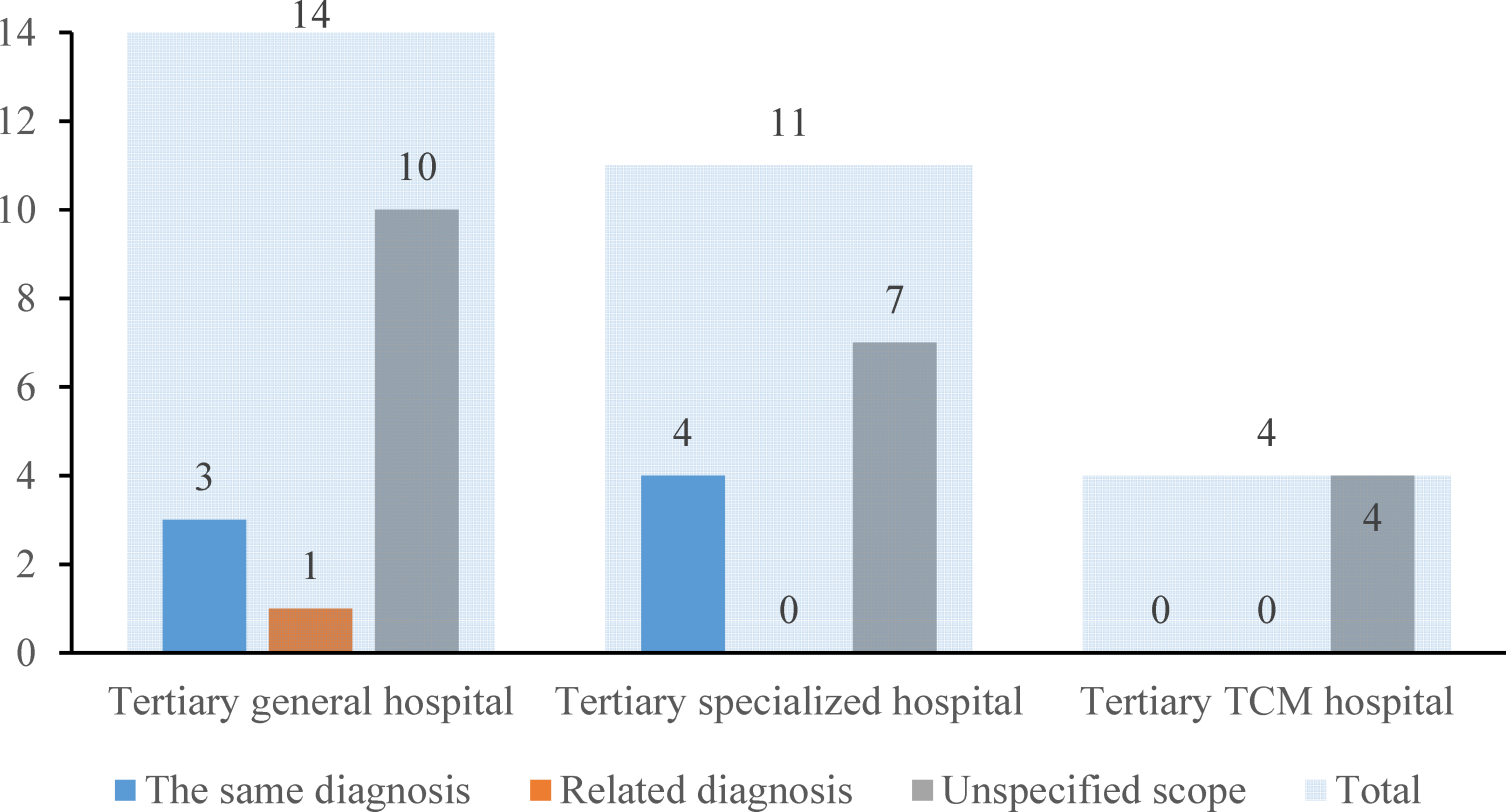
Institution distribution on prerequisites for diagnosis of previous visits (n=29). The light blue shade indicates the sum in each type of hospital. The blue, orange, and gray columns indicate the number of hospitals that required the patients to have the same diagnosis, related diagnosis, and unspecified scope, respectively. TCM: traditional Chinese medicine.

The prerequisites for diagnosis of previous visits were divided into 3 categories: the same diagnosis, related diagnosis, and unspecified scope. Figure 4 shows that 72.4% (21/29) of the nonprofit hospitals imposed no restrictions on the diagnosis of previous visits. There were 3 general hospitals and 4 specialized hospitals that required the diagnosis of the previous visit to match the current diagnosis. One general hospital required the diagnosis of the previous visit to be related to the current diagnosis. TCM hospitals had no restrictions regarding previous diagnosis.

#### Prerequisites for Visit Interval

**Figure 5.**
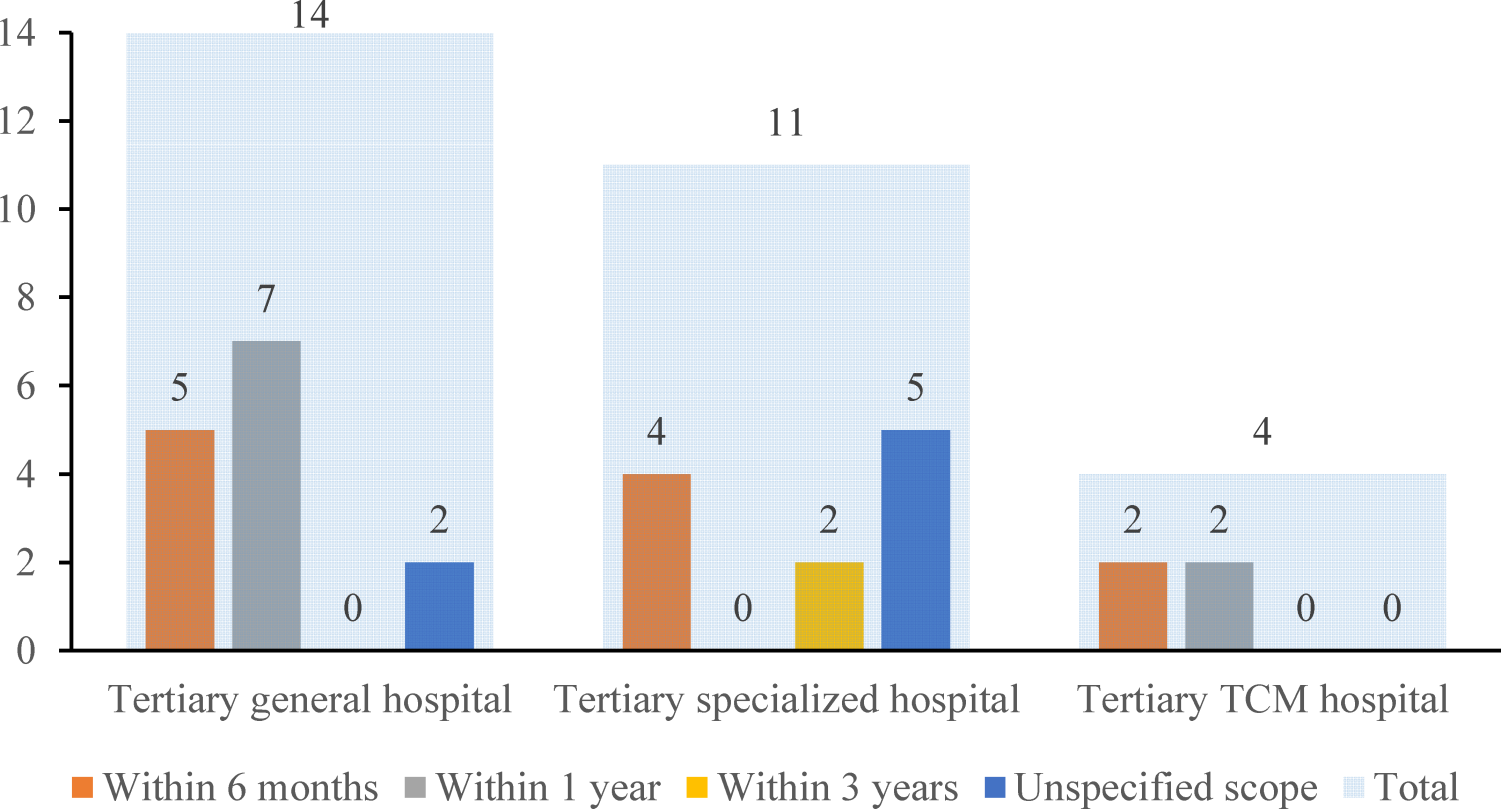
Institution distribution on prerequisites for visit interval (n=29). The light blue shade indicates the sum in each type of hospital. The orange, gray, yellow, and blue columns indicate the number of hospitals that required the visit interval to be within 6 months, within 1 year, within 3 years, and unspecified scope, respectively. TCM: traditional Chinese medicine.

The prerequisites for visit interval were divided into 4 categories: within 6 months, within 1 year, within 3 years, and unspecified scope. Figure 5 shows that 37.9% (11/29) of the hospitals restricted the interval to be within 6 months, 31.0% (9/29) of the hospitals restricted it to be within 1 year, and 6.9% (2/29) of the hospitals restricted it to be within 3 years. The 2 specialized hospitals that restricted the visit interval to be within 3 years were both pediatric hospitals. Among the 29 hospitals, 2 general hospitals and 5 specialized hospitals did not have any restrictions regarding the visit interval.

### Prerequisites for Patient Assessment in Private, For-Profit Medical Institutions in Beijing

Most of the other 7 private, for-profit institutions did not have any restrictions on the above 3 dimensions. At least 1 secondary general hospital restricted the previous diagnosis to be the same as the current one. This hospital, along with a first-class TCM hospital, required the visit interval to be within 6 months. The other institutions did not have any requirements on the above 3 dimensions to assess whether patients could get telemedicine services. Overall, there were no significant disparities in prerequisites for patient assessment across different types of private, for-profit medical institutions.

### Considerations for the Patient Eligibility Assessment

As mentioned by the National Health Commission, patients were required to provide relevant proof of diagnosis from past visits, with no restrictions on the visit interval or the scope of the previous visit location (ie, institution or department) [14]. Physicians providing telemedicine services were responsible for assessing patients. However, in practice, most institutions set requirements in the system for the location of previous visits and the visit interval while having fewer requirements on the diagnosis in the first assessment stage. The reason for imposing such requirements was that obtaining medical records from their own institution was more convenient. The visit intervals were set according to the typical disease progression timelines of the main diseases treated in each department. When telemedicine services restricted the scope of the previous visit location and the visit interval, it became easier to determine eligibility by referencing the patient’s diagnosis from a specific institution at a particular point in time.

As shown in Figure 3, 26 institutions required a record of previous visits within their own institution. As they declined to acknowledge or accept previous visit records originating from external institutions, we obtained the following reasons (Table 4) from these institutions through interviews. The primary reason (19 interviewees) was distrust of the medical results provided by other institutions. The second most frequently mentioned reason (18 interviewees) was that it was more challenging to handle medical disputes involving other institutions once they happened. Among these 19 and 18 institutions, only 1 specified that the previous visit must be with the same physician, and 2 required that the previous visit must be within the same department. The third most frequently mentioned reason (10 interviewees) was the system’s limitation to access data from other institutions. Notably, these 10 services did not impose additional restrictions regarding whether the previous visit was with the same physician or within the same department. Acceptance was granted as long as the patient had any history of visits within their own institution.

**Table 4.** Distribution of reasons for service restrictions in 26 institutions.

Reasons	Institutions, n (%)	Institutions with prerequisites for the scope of previous visit location, n		
		Within the same institution	Within the same department	Within the same physician
Distrust in the medical results provided by other institutions	19 (73.1)	16	2	1
Difficulties in handling medical disputes involving other institutions once medical disputes happen	18 (69.2)	15	2	1
Limited access to data in other institutions through information technology system	10 (38.5)	10	0	0

After the system automatically administered the first assessment stage, physicians would still terminate the telemedicine services in the second stage. Through interviews with the contacts of 36 hospitals, the main reasons for the termination included the patient’s condition (n=22, 61.1%), mismatch due to the rules preestablished in the telemedicine system (n=15, 41.7%), and inability to provide the services that the patient hopes to receive (n=13, 36.1%) (Table 5). The proportion of service termination by physicians could be controlled within 2% of the total amount of telemedicine services.

**Table 5.** Institution distribution on reasons for the termination of telemedicine services (n=36).

Reasons	Institutions, n (%)
The patient’s condition is not suitable for telemedicine services (eg, abdominal pain, trauma, the blood pressure of the hypertensive patient has become so high that physicians cannot refill prescriptions for the patient)	22 (61.1)
Mismatch due to the rules preestablished in the telemedicine system (eg, optometry physicians categorized as ophthalmologists in the system cannot provide services to cataract patients)	15 (41.7)
Inability to provide the services the patient hopes to receive (such as prescribing medications, conducting special examinations)	13 (36.1)
Communication problems due to network issues	7 (19.4)
Not meeting the visit interval required by medical insurance	7 (19.4)
Time conflicts with physicians	5 (13.9)
Insurance fraud committed by patients	2 (5.6)
Unknown	2 (5.6)

## Discussion

### Principal Findings

This study investigated the current practice of patient eligibility assessment for telemedicine services in Beijing, elucidated institutions’ rationale for adopting such approaches, analyzed their differences from national policies, and proposed policy recommendations. First, most nonprofit, tertiary hospitals automatically assessed patient eligibility by the system in the first stage based on the scope of previous visit location, diagnosis of previous visits, and visit interval. Second, compared with national policies, nonprofit hospitals had stricter requirements on the scope of previous visit locations and more lenient requirements on the diagnosis of previous visits. On the other hand, private, for-profit institutions exhibited more relaxed requirements in all dimensions. Third, 2 main reasons for the discrepancies between policy and practice were distrust in the medical results provided by other institutions and difficulties in handling medical disputes involving other institutions once medical disputes happened. Based on the discrepancies, our findings recommend enhancing policy clarity, relaxing telemedicine regulation for new patients, and strengthening telemedicine supervision to improve telemedicine services.

### Differences in Prerequisites for Patient Assessment

The quantitative data revealed differences across prerequisites for patient eligibility assessment among various types of medical institutions. Nonprofit hospitals had stricter requirements than private, for-profit institutions on patient eligibility assessment in the above 3 prerequisites. TCM hospitals, which had the highest average volume of telemedicine services, neither restricted prerequisites for the scope of previous visit location to specific departments or physicians nor imposed specific restrictions on patients' diagnoses of previous visits. The main reasons for the flexibility of these hospitals were the holistic view [27] and the personalized treatment approach in TCM, which relies on each physician’s own professional judgment [28]. The general hospital with the highest service volume restricted the current diagnosis to be related to the diagnosis of previous visits, considering the potential variations in the scope of diagnosis and treatment for certain departments across different hospitals. The prerequisites for the visit interval were primarily determined by the disease characteristics among the majority of the patients. We have observed that only 2 hospitals, both pediatric, adopted a 3-year visit interval. As this may be related to the rapid growth of pediatric outpatient visits and relatively long waiting times [29,30], we hypothesize these hospitals seek to transition suitable cases to telemedicine services when appropriate. Most of the other private, for-profit medical institutions did not impose restrictions on the above 3 dimensions.

The qualitative data explained the discrepancies between policy and practice. As for the results of quantitative analysis, the prerequisites for the scope of previous visit location and diagnosis of previous visits showed the gap from policy requirements, while the prerequisites for visit interval were not mentioned in the policies. Our qualitative analysis demonstrated the reasons for barriers to the acceptance of patients based only on external institution visit records. Since determining whether a diagnosis from previous visits aligned with the requirements of the current institution usually involves professional judgment, this assessment cannot be made automatically by the system. Findings from the interviews revealed that the main reasons for the termination of telemedicine service included the patient’s condition not being suitable for telemedicine services and mismatches preventing physicians from providing treatment to patients with specific diagnoses.

### Reasons for the Discrepancies Between Policy and Practice in Nonprofit Hospitals

The results specified that 2 main reasons had caused the discrepancy in the scope of the previous visit location between policy and practice. The first reason was distrust in the medical results provided by other institutions, and the second reason was difficulties in handling medical disputes involving other institutions once they happened. In practice, service quality and safety were the primary factors that nonprofit hospitals considered. Telemedicine services were only a supplement for traditional brick-and-mortar medical institutions [31]. Therefore, nonprofit hospitals were more cautious in assessments by the system, limiting the scope of previous visit locations to their hospital.

Most nonprofit hospitals did not have restrictions on the diagnosis of previous visits in the first assessment stage. Due to the ambiguity of national policies regarding the requirements of a patient’s diagnosis for telemedicine services, it was difficult to quickly assess patient eligibility (except for a few hospitals that limit the scope of previous visit locations to the same department). The physicians always assessed patient eligibility related to the diagnosis of previous visits in the second stage and would terminate services if the patient’s condition was not suitable for telemedicine.

Another frequently mentioned reason for differences was barriers to accessing patients’ medical information in other institutions. The efficiency of assessment automatically performed by the system was higher than that manually performed by the physician. The system automatically limited the scope of previous visit locations to the same hospital to accommodate the large volume of telemedicine services in Beijing. However, national policies allowed patients to access telemedicine services after their first visit to a brick-and-mortar institution, regardless of the scope of the previous visit location. To be consistent with the policy’s requirements, it is necessary to achieve interconnection among health systems in the region. Health information systems, including interoperability with electronic medical records, are a challenge in developing telemedicine services in other countries [32]. Systems that help with initial assessments would quickly capture patients’ records at other institutions, reducing physicians’ time and effort on evaluation. Beijing had already made a preliminary attempt in this regard. Physicians in 140 secondary and tertiary medical institutions had access to 181 test results and 300 imaging test results in these institutions, and the results were mutually recognized [33]. Access to test results laid the foundation for access to health information records in the future.

### Reasons for the Discrepancies Between Policy and Practice in Private, For-Profit Institutions

Private, for-profit medical institutions were more lenient than nonprofit hospitals in assessing patient eligibility. Most of the private, for-profit institutions did not set requirements apart from the policy requirements. However, there was a possibility of relaxing the policy requirements in practical work. Most private, for-profit medical institutions initiated by enterprises took telemedicine services as their primary development direction. When enterprises were the main initiators of medical institutions, they focused on increasing operating income and achieving profitability [18]. Patient eligibility for telemedicine services was always assessed manually by physicians in these institutions, and the actual assessment criteria may have been more permissive than policy requirements. In addition, the private, for-profit medical institutions mainly provided psychological and traditional Chinese medical services, and the telemedicine services were also concentrated on psychological counseling and prescriptions. Physicians focused more on the patients’ needs of current telemedicine visits rather than the prerequisites for the scope and diagnosis of previous visits.

### International Telemedicine Policies

Telemedicine was once limited only to rural or remote communities but is now increasingly used to expand the geographic reach of health care services and improve access to care [34]. According to the 2023 report on the state of digital health in the World Health Organization (WHO), 78.4% (40/51) of the WHO Europe Member States directly addressed telehealth in their policies or strategies [35]. While the use of telemedicine was generally allowed in most countries, the eligibility requirements for telemedicine service users had changed due to the in-person visits sharply declining during the COVID-19 pandemic. Before March 2020, 36.7% (11/30) of the Organization for Economic Co-operation and Development countries allowed telemedicine only for patients who had an in-person visit in the past, but after March 2020, only 20.0% (6/30) of the countries imposed the requirement [36]. Unlike most Organization for Economic Co-operation and Development countries, in China, an initial face-to-face meeting between the physician and the patient is still needed before telemedicine services, except for patients with COVID-related symptoms.

The establishment of patient eligibility requirements was primarily intended to ensure the safety of patients engaging in telemedicine services. The comprehensive framework designed by the WHO to enhance and streamline telemedicine services included a clinical risk assessment process. The process specified which services were safe for the patients to use independently and which needed expert medical support [37]. We observed that countries employed 2 methods to ensure safety: establishing telemedicine service lists or relying on physicians’ professional judgment. The United States Drug Enforcement Administration allowed patients to obtain prescribed medications through telemedicine appointments, without requiring an initial in-person medical evaluation, except for prescribing controlled medications to new patients [38]. In Japan, it was the physicians’ sole responsibility to determine whether telemedicine service was appropriate and safe [39]. Hungary not only restricted the types of telemedicine services but also mandated that physicians make the final diagnosis [39]. In China, the policy has not yet explicitly outlined a list of specific service types for telemedicine. At present, physicians play a key role in determining the services offered to patients and ensuring their safety. Experiences in other countries shed light on how China could refine its telemedicine policies.

Providing telemedicine services to new patients is expected to become a major trend in future health care, with ensuring patient safety as an essential component. A more profound understanding of what visits are appropriate for telemedicine and better triaging of visit types are crucial steps to maintain the quality of care [40]. However, the legal responsibilities of physical medical institutions and physicians regarding issues such as medical risk have not been fully clarified [41]. Supported by the telemedicine service list, the challenges and work-related stress for physicians can be alleviated.

### Suggestions for Future Improvement

According to the above analyses, telemedicine policies could be optimized in the following aspects: first, the ambiguous parts of the policies could be clarified. Second, the content of telemedicine services for new patients could be added to the policies. Third, policies for telemedicine services would be made for nonprofit and private, for-profit institutions, respectively.

Explicit policies would help providers implement the requirements stipulated in the policies and make determinations about the applicability of telemedicine services more easily [42]. Although several policy interventions had been implemented at the national level, there was still a lack of specific service standards to direct frontline service delivery [31]. Specific disease names or diagnoses were not defined. A guideline could be made, including a comprehensive list of symptoms or diagnoses provided through telemedicine. Guidelines could also be considered when the most recent in-person visit occurs [42]. For example, the American Medical Association made the Telehealth Services Covered by Medicare and Included in the Current Procedural Terminology Code Set [43]. The United States Drug Enforcement Administration stated that a patient could receive a 6-month supply of buprenorphine through telehealth services; further prescriptions would require an in-person visit [38].

There were already some mature precedents for policymakers to refer to, allowing telemedicine health services for new patients. In the United States, telemedicine services were allowed reimbursement and expanded coverage for telehealth services to new and established patients from March 2020 [44,45]. An in-person visit within 6 months of an initial mental telehealth service is not required through September 30, 2025 [46]. After March 2020, 7 countries relaxed the prerequisite that patients were only allowed to have teleconsultations with physicians they had already consulted in person [36]. In this study, some institutions were found to provide telemedicine services for new patients. The service volume of 1 private, for-profit institution in 2023 was 134,715, ranked third among 36 institutions in this study. In the supervision process, it was found that this institution focused on psychological services and provided telemedicine services for new patients. However, no significant incidents had affected medical quality and patient safety. In the United States, 80% of the mental health treatment facilities that accepted new clients could offer telehealth services [47]. Telemental health use also increased substantially and globally during the pandemic and will remain in use for the foreseeable future [48,49].

In China, from the demand-side perspective, the conditions for opening up telemedicine services for new patients have been met under certain circumstances. Telemedicine services provide access to high-quality medical care, free up resources in overcrowded public hospitals [50], and also help to alleviate the uneven distribution of medical resources [51]. Telemedicine services before and during the COVID-19 pandemic had enhanced the delivery of and access to health care services [52] and increased satisfaction compared with usual in-person care [53]. They allowed unprecedented accessibility for those with mobility or geographical restrictions [54]. Considering the advantages of telemedicine services, there was also a need for new patients to access them.

However, to deal with the potential risks of providing services for new patients, the following measures could be considered. First, standardize the scope of telemedicine services (eg, ordering examinations or tests) for new patients to ensure medical safety [39]. Experts from various specialties would be engaged to develop a telemedicine service list for new patients, detailing diagnostic criteria, disease stages, and specific service items, as well as prohibiting telemedicine in unsuitable cases. Furthermore, developing an interoperable electronic medical record system that enables doctors to access patients’ previous medical records and test results would enable physicians to understand patients’ conditions more accurately. Second, select the diseases for which the diagnostic and treatment schemes are relatively mature with a high frequency of visits (eg, diseases of dermatology and psychology [47,55]) to meet the patients’ needs for privacy protection and reduce the round-trip travel time [50]. Hospitals with high-quality telemedicine services could pilot telemedicine for new patients, starting with 2‐3 medical specialties that carry low clinical risk. Third, improve the requirements for the professional experience and professional title of physicians who can provide telemedicine services for new patients. Finally, limit the scope of services covered by medical insurance funds. If the patient only needs a medical examination and related consultation, the patient would pay the relevant fees at his or her own expense.

The supervision policies of the services need to be formulated separately due to different initiators of institutions to ensure the quality and safety of services. The primary purpose of telemedicine services initiated by nonprofit hospitals is to expand the scope and intensity of hospital services, while the purpose of telemedicine services initiated by private, for-profit institutions is to increase the number of patients and gain benefits [18]. For nonprofit medical institutions with strict management, policies can appropriately relax the requirements of patient eligibility for telemedicine services and even prioritize providing telemedicine services for new patients. For private, for-profit institutions, policies need to restrict the types of telemedicine services provided by private, for-profit institutions and raise the requirements for the qualifications of physicians in telemedicine services. In addition, since most private, for-profit institutions manually assess patient eligibility by the physician, additional regulation is required (eg, making data of the service process traceable) to ensure the safety and quality of telemedicine services [56]. During regulation, the Supervision Platform can proactively monitor adverse events and quality deficiencies and automatically submit the above results to both medical institutions and the Center on a daily basis. The Center can also organize third-party experts to conduct annual inspections, and institutions that fail to meet the standards will be prohibited from providing telemedicine services.

### Limitations

The 36 institutions selected for this study included those with high service quality and large service volumes, but those with lower service volume or quality were excluded. This is because, during routine inspections conducted by the Center, we found that these institutions often lacked stable management systems and assessment processes, making it challenging to reasonably compare their assessment processes with national policy. Therefore, these institutions were excluded from this study, making it impossible to characterize their follow-up criteria.

Additionally, there would be potential social desirability bias in interviews [57]. In an effort to minimize this potential bias, before conducting the interviews, we informed participants about the anonymization of data and ensured that their specific responses would not be disclosed; only the statistically analyzed group information would be made public. We also cross-validated interview results against previous inspection findings to ensure the authenticity of the results.

Finally, the study may have missed considerations and insights from policy formulation and regulatory constraints. Incorporating government perspectives could have enhanced the comprehensiveness of the study. We plan to submit our research results to the relevant government departments to inform policymakers about policy implementation and help elucidate the underlying reasons for the discrepancies between telemedicine policy and actual practice.

### Conclusions

This pioneering multicenter, mixed methods study innovatively analyzed patient eligibility for telemedicine services in China, addressing the current gap in telemedicine policy research. The findings faithfully reflect the practices of the patient eligibility assessment process for telemedicine services in Beijing. Furthermore, by integrating quantitative and qualitative analyses, the study uncovered the underlying reasons behind discrepancies between institutional practices and national policy requirements, providing valuable insights about current implementation challenges. These results suggest potential improvements, including relaxing the regulation for new patients’ access to telemedicine services, enhancing service delivery processes, and strengthening the robustness of the telemedicine supervisory framework.

## Supplementary material

10.2196/75964 Multimedia Appendix 1 Regulatory compliance assessment of telemedicine services: 34-item compliance monitoring metrics from the Beijing Telemedicine Supervision Platform.

10.2196/75964 Multimedia Appendix 2 Survey questionnaire for patient eligibility assessment in telemedicine services.

10.2196/75964 Multimedia Appendix 3 Interview outline and representative interview excerpts for institutional considerations of the patient eligibility assessment.

10.2196/75964 Checklist 1 Checklist for reporting mixed methods research (MMR) studies.

## Abbreviations

TCM: traditional Chinese medicine
WHO: World Health Organization
