# Supplementary material for: Bridging Policy and Practice in Telemedicine Follow-Up Identification: Multicenter Mixed Methods Study in Beijing
Source: JMIR Hum Factors. 2025 Dec 19;12:e75964. doi: 10.2196/75964 (PMC12716420; doi:10.2196/75964)
Supplement: Multimedia Appendix 1 [file humanfactors-v12-e75964-s001.doc]

**Multimedia Appendix 1.** **Regulatory compliance assessment of telemedicine services: 34-item compliance monitoring metrics from the Beijing Telemedicine Supervision Platform.**

| Category | Subcategory | No. | Criteria |
| --- | --- | --- | --- |
| Qualification | Medical institution qualifications | 1 | Expired telemedicine service license. |
|  |  | 2 | Expired practice license of the physical medical institution upon which the telemedicine service is established. |
|  |  | 3 | Continuation of telemedicine services during suspension or delayed verification of the affiliated physical medical institution. |
|  |  | 4 | Failure to validate the affiliated physical medical institution within the scheduled period. |
|  |  | 5 | Continuation of telemedicine services after revocation of the physical medical institution’s practice license. |
|  |  | 6 | A mismatch between medical institution categories and telemedicine service authorization. |
|  |  | 7 | Telemedicine services exceeding the range of medical services of the physical medical institution. |
|  |  | 8 | Expired collaboration agreement with third-party telemedicine system providers. |
|  | Physician qualifications | 9 | Unregistered physicians in the national electronic physician registration system. |
| Operation | Diagnosis and treatment behavior | 10 | Absence of diagnostic documentation. |
|  |  | 11 | Missing timestamp for the current medical visit. |
|  |  | 12 | Omission of medical department information. |
|  |  | 13 | Lack of patient name documentation. |
|  |  | 14 | Missing patient gender information. |
|  |  | 15 | Absence of patient identification number. |
|  |  | 16 | Invalid identification number format. |
|  |  | 17 | Missing patient contact details. |
|  |  | 18 | No recorded current patient address. |
|  |  | 19 | Absence of guardian’s information for patients under six years old. |
|  |  | 20 | Unspecified follow-up visit status. |
|  |  | 21 | Missing consultation details. |
|  |  | 22 | No information about the hospital that initiated the telemedicine consultation. |
|  |  | 23 | No medical institution information for previous visit. |
|  |  | 24 | Unqualified consulting physicians. |
|  | Risk Warnings for Patients | 25 | Failure to provide patient risk warnings. |
|  |  | 26 | A lack of signed informed consent forms. |
| Supervision | Prescriptions | 27 | Prescriptions without pharmacist review. |
|  |  | 28 | Documented patient age exceeding 120 years. |
|  |  | 29 | Incomplete prescription details (drug name, specifications, quantity, usage, or dosage). |
|  |  | 30 | Single prescription containing over five medications (excluding herbal prescriptions). |
|  | Narcotic and psychotropic drugs | 31 | Prescription of psychotropic medications without authorization. |
|  |  | 32 | Prescription of narcotic medications without authorization. |
|  | On-site inspection | 33 | Active administrative penalties on the telemedicine institution. |
|  |  | 34 | Regulatory penalties imposed on the telemedicine institution. |
