# Supplementary material for: Bridging Policy and Practice in Telemedicine Follow-Up Identification: Multicenter Mixed Methods Study in Beijing
Source: JMIR Hum Factors. 2025 Dec 19;12:e75964. doi: 10.2196/75964 (PMC12716420; doi:10.2196/75964)
Supplement: Multimedia Appendix 2 [file humanfactors-v12-e75964-s002.doc]

**Multimedia Appendix 2.** Survey questionnaire for patient eligibility assessment in telemedicine services

Thank you for participating in this research initiative. This 5-item survey aims to clarify the approaches and patient eligibility assessment process for telemedicine services. Your responses will directly inform policy recommendations to optimize telemedicine services frameworks.

1. What approach does your institution use to assess patient eligibility for telemedicine services in your institution? (Single choice)

A. Automatically by the system

B. Manually by the physician

1. According to the scope of previous visit location, which of the following meets the requirements for telemedicine services in your institution? (Single choices)
2. Previous visit in person was to any department of your institution
3. Previous visit in person was to the same department of your institution
4. The last visit was with the same doctor
5. No specified scope
6. Other (please specify)
7. According to the diagnosis of previous visits, which of the following meets the requirements for telemedicine services in your institution? (Single choices)
8. The same diagnosis as the previous in-person visit
9. Related diagnosis with the previous in-person visit
10. No specified scope
11. Other (please specify)
12. According to the visit interval between the previous and current visits, which of the following meets the requirements for telemedicine services in your institution? (Single choices)
13. Within 6 months
14. Within 1 year
15. Within 3 years
16. No specified scope
17. What actions will be taken if the patient does not meet the requirements? (Multiple choices)
18. The system blocks the request, preventing telemedicine services
19. The attending physician informs the patient to visit in person
20. The attending physician helps the patient make an in-person appointment
21. Other (please specify)
