# Supplementary material for: Bridging Policy and Practice in Telemedicine Follow-Up Identification: Multicenter Mixed Methods Study in Beijing
Source: JMIR Hum Factors. 2025 Dec 19;12:e75964. doi: 10.2196/75964 (PMC12716420; doi:10.2196/75964)
Supplement: Multimedia Appendix 3 [file humanfactors-v12-e75964-s003.doc]

**Multimedia Appendix 3.** Interview outline and representative interview excerpts for institutional considerations of the patient eligibility assessment.

The interview outline for institutional considerations of the patient eligibility assessment is presented as follows:

We sincerely appreciate your valuable participation in our initial survey on telemedicine service implementation. We are conducting follow-up interviews with selected institutional representatives to further validate critical findings and clarify institutional considerations of prerequisites for patient assessment and growth strategies details. The interview takes approximately 15-20 minutes.

1. Does your institution support card-free online billing through the medical insurance? What’s the difference between synchronous and asynchronous telemedicine services with medical insurance?
2. How does your institution set the prerequisites for visit intervals? Please choose between:
3. Based on the patient’s previous visits stored in the electronic medical record system, the system automatically decides whether the visit interval is long enough.
4. Based on the information (medical records from other institutions) the patient uploaded, physicians manually decide whether the visit interval is long enough.

What factors were considered in establishing prerequisites for visit intervals?

1. What is the rate of physician-initiated suspension of telemedicine services? What are the specific reasons?
2. “Follow-up patients” must be “patients who have visited any department within the same hospital,” according to some hospitals. However, the Minister of Health says “follow-up” patients must be “patients with a clear diagnosis and medical records seeking medical service for the same diagnosis.” What do you suppose is the cause of this difference? What may lead to the discrepancy, based on your own experience?
3. If telemedicine services are permitted for new patients, will your institution consider piloting a specific department or opening all departments? If you decide to pilot, which department will be prioritized? Why?

**Table 1.** Representative interview excerpts of reasons for service restrictions

| Reasons | Excerpts |
| --- | --- |
| Distrust in the medical results provided by other institutions | “Well, we don’t trust external medical records because it's hard to verify their quality.”  “Sure. Sometimes a patient comes in with an ultrasound report from another hospital that shows nothing concerning, but when we do the scan here, we might pick up on something different.” |
| Difficulties in handling medical disputes involving other institutions once medical disputes happen | “Yeah, we had a case where a patient came to us for leg pain after receiving treatment at another hospital. It was eventually diagnosed as cancer here. The patient later complained that we delayed the diagnosis. In such situations, it's incredibly difficult to determine where the actual delay occurred—at our facility or the previous one.”  “In one case, a patient received medication via our telemedicine platform based on a prescription from another hospital that had lacked the drug in stock. The patient subsequently suffered a sudden death. Ultimately, it was unclear where the responsibility lay.” |
| Limited access to data in other hospitals through information technology system | “Because different hospitals use different technology companies to build their systems, it's really hard to get them to talk to each other. The technology doesn't match up, and there are always security rules blocking the way."”  “To keep patient info safe and secure, we just don't want to connect our system to others. It's not worth the risk.” |

**Table 2**. Representative interview excerpts of reasons for the termination of telemedicine services

| Reasons | Excerpts |
| --- | --- |
| The patient’s condition is not suitable for telemedicine services (e.g., abdominal pain, trauma, the blood pressure of the hypertensive patient has become so high that physicians cannot refill prescriptions for the patient, etc.) | “In cases where patient medical records are incomplete, we terminate telemedicine services to ensure safety. Patients are referred to in-person clinics for comprehensive assessment. Telemedicine may be reinstated following a confirmed diagnosis.”  “Emergency conditions, such as acute abdominal pain, are excluded from our telemedicine protocol. These cases are immediately redirected to emergency departments.” |
| Mismatch due to the rules pre-established in the telemedicine system (e.g., optometry physicians categorized as ophthalmologists in the system cannot provide services to cataract patients.) | “The patient got confused by the medical terms when booking. They actually wanted treatment for their cataracts, but ended up making an appointment with an ophthalmologist who only does vision tests.”  “In our hospital, strict specialty division (e.g., Orthopedics vs. Sports Medicine) leads to patient confusion, especially when such division is uncommon in other hospitals.” |
| Unable to provide the services the patient hopes to receive (such as prescribing medications, conducting special examinations, etc.) | “The patient wants a prescription for some medications, but they're not allowed to be prescribed online. For example, if a patient asks for sleeping pills, we can't prescribe those through our online hospital for safety reasons.”  “Unlike some hospitals, we don't prescribe herbal medications, so patients who want those would need to cancel their appointment here and go elsewhere.” |
| Communication problems due to network issues | “We had patients who missed their scheduled online visits and were unreachable. They were unable to be contacted throughout the entire day.”  “Occasionally, the telemedicine mini-program experiences technical problems. If a patient fails to connect at their scheduled time due to such issues, we automatically process a refund for the missed appointment.” |
| Not meet the visit interval required by medical insurance | “The patient wants to use insurance for this medication, but there's a time restriction on refills. It's too soon according to the insurance rules to get a refill.” |
| Time conflict with doctors | “The doctor is still in surgery and won't be able to make the online consultation.” |
| Insurance fraud committed by patients | “The patient is using someone else's insurance card, not their own. So we have to stop the online visit.” |
| Unknown | “Sorry, we don't have that figure available right now since we haven't done the detailed analysis.” |
