# Supplementary material for: Bridging Policy and Practice in Telemedicine Follow-Up Identification: Multicenter Mixed Methods Study in Beijing
Source: JMIR Hum Factors. 2025 Dec 19;12:e75964. doi: 10.2196/75964 (PMC12716420; doi:10.2196/75964)
Supplement: Checklist 1 [file humanfactors-v12-e75964-s004.doc]

**Checklist 1. Checklist for reporting mixed methods research (MMR) studies.**

| Category | Checklist items | Position in the paper |
| --- | --- | --- |
| Rational and description of mixed method research design | Provide a clear statement of the study purpose | Page 2 in Introduction- Aims and Objectives |
|  | Explicitly describe the Mixed method research design in accordance with Creswell’s (2015) typology and use a diagram to illustrate the relationship and sequence of qualitative and quantitative research components | Page 2-4 in Methods- Overview |
|  | Justify why the Mixed method research design is appropriate for meeting the study purpose | Page 3 in Methods-Overview |
| Transparency in describing method details | Describe the study population(s) and sample (s; e.g., who, what, how many) | Page 4 in Methods- Institution Recruitment |
|  | Describe the sampling procedures (including inclusion and exclusion criteria, recruitment) | Page 4 in Methods- Institution Recruitment |
|  | Describe qualitative data collection processes (how often data were collected, who collected the data, what kind of data collection instruments were used, how data were recorded—e.g., notes, transcripts) | Page 5 in Methods- Data Collection and Management Procedures- Semi-structured Interview |
|  | Describe quantitative data collection processes (how often data were collected, who collected the data, what kind of data collection instruments were used measurements, validity/reliability) | Page 4 in Methods-Data Collection and Management Procedures |
|  | Describe qualitative data analysis processes (coding, single or multiple coders, replication logic, credibility) | Page 5 in Methods-Data Analysis |
|  | Describe quantitative data analysis procedures (missing data and how they are handled, statistical tests used) | Page 5 in Methods-Data Analysis |
| Integration of qualitative and quantitative research components | Interpret qualitative analysis results with appropriate quotes if necessary | Page 10-11 in Results- Considerations for the Patient Eligibility Assessment  Appropriate quotes listed in Multimedia Appendix 3 |
|  | Interpret quantitative analysis results in consideration of statistical significance, selection bias, and threats to validity | Page 8-10 in Results- Prerequisites for Patient Assessment in Non-profit Tertiary Hospitals in Beijing |
|  | Compare qualitative and quantitative results | N.A., quantitative and qualitative research were conducted sequentially to address different aspects of the study: quantitative research identified discrepancies between practice and policy, while qualitative research explained these differences. |
|  | Address divergences and inconsistencies between qualitative and quantitative results | N.A., same as above. |
